# Gestational and congenital syphilis in a Brazilian harbor city: A population-based study of prevalence, determinants, and adverse neonatal outcomes

**DOI:** 10.1016/j.bjid.2026.105969

**Published:** 2026-09-30

**Authors:** Mariana Rodrigues Jacomini Alarcon, Paulo Neves, Davi Castor da Silva, Luiz Alberto Amador Pereira, Maria De La Ó Ramallo Verissimo, Rosa Moreira, Maíra Barreto Malta

**Affiliations:** a Postgraduate Program in Health Sciences, Universidade do Oeste Paulista (UNOESTE), Presidente Prudente, SP, Brazil; b Centre for Global Child Health, The Hospital for Sick Children (SickKids), University of Toronto, Toronto, Ontario, Canada; c School of Medicine, Universidade do Oeste Paulista (UNOESTE), Guarujá, SP, Brazil; d Department of Maternal-Child and Psychiatric Nursing, School of Nursing, University of São Paulo (USP), São Paulo, SP, Brazil; e University of Coimbra, UICISA:E, ESEUC, Portugal

**Keywords:** Syphilis, Syphilis, congenital, Infectious disease transmission, vertical, Infant mortality, Prenatal care

## Abstract

**Background:**

Gestational and congenital syphilis remain major public health concerns, particularly in low- and middle-income countries, with substantial impacts on neonatal health. This study estimated the prevalence of gestational syphilis and the birth prevalence of congenital syphilis, identified factors associated with gestational syphilis, and evaluated the association between congenital syphilis and adverse neonatal outcomes in Guarujá, São Paulo, Brazil.

**Methods:**

This cross-sectional study was nested within the population-based Guaru-yá Birth Cohort and included 2,217 hospital births from April 2024 to April 2025. Gestational syphilis was defined by reactive treponemal and non-treponemal tests during pregnancy or delivery, considering diagnosis and treatment during the current pregnancy; adequately treated women were not classified as cases. Congenital syphilis was defined according to clinical, laboratory, or epidemiological criteria compatible with vertical transmission. Adjusted Prevalence Ratios (PR) and 95% Confidence Intervals (95% CI) were estimated using Poisson regression with robust variance.

**Results:**

Gestational syphilis prevalence was 12.8% (95% CI: 11.4–14.2), and congenital syphilis birth prevalence was 5.8% (95% CI: 4.8–6.8). Higher gestational syphilis prevalence was observed among women with low maternal education (PR = 2.47; 95% CI: 1.43–4.29), living in poorest households (PR = 1.46; 95% CI: 1.07–2.00), who did not cohabit with a partner (PR = 1.83; 95% CI: 1.45–2.31), who smoked during pregnancy (PR = 1.75; 95% CI: 1.35–2.27), and used illicit drug during pregnancy (PR = 1.70; 95% CI: 1.26–2.28). Women aged ≥ 35 years showed lower prevalence of gestational syphilis compared with those aged 20–34 years (PR = 0.67; 95% CI: 0.47–0.97). Congenital syphilis remained independently associated with fetal or neonatal death (PR = 3.25; 95% CI: 1.74–6.07).

**Conclusions:**

Gestational syphilis was strongly associated with socioeconomic vulnerability and behavioral factors. Congenital syphilis remained independently associated with fetal or neonatal death, reinforcing the need for early prenatal access, repeated screening, timely maternal and partner treatment, and strengthened surveillance to reduce vertical transmission.

## Highlights

• Gestational syphilis prevalence reached 12.8% in a Brazilian harbor city.
• Congenital syphilis birth prevalence was 5.8% in the study population.
• Social vulnerability was strongly associated with gestational syphilis.
• Smoking and illicit drug use were independently associated with gestational syphilis.
• Congenital syphilis was independently associated with fetal or neonatal death.

## Introduction

Syphilis is a sexually transmitted bacterial infection caused by *Treponema pallidum* that can be vertically transmitted from mother to fetus during pregnancy or at delivery.

Gestational syphilis refers to maternal syphilis diagnosed during pregnancy, childbirth, or the puerperium, whereas congenital syphilis results from fetal infection through vertical transmission and remains an important preventable cause of fetal and neonatal morbidity and mortality worldwide.^1^^,^^2^

When not adequately diagnosed and treated during prenatal care, syphilis may result in miscarriage, stillbirth, prematurity, low birth weight, neonatal death, and permanent sequelae, including deafness, blindness, and neurological impairment.^2^^,^^3^ Vertical transmission of *Treponema pallidum* may occur at any stage of pregnancy and remains strongly associated with delayed diagnosis, untreated or inadequately treated maternal infection, and inadequate prenatal care.^2^^,^^4^

Despite the availability of accurate diagnostic tests and effective treatment with penicillin, congenital syphilis remains a major global public health problem. It is estimated that maternal syphilis affects between 1 and 1.8 million pregnancies annually worldwide, resulting in more than 300,000 fetal and neonatal deaths, in addition to hundreds of thousands of cases of prematurity, low birth weight, and congenital infection-related complications. This burden is concentrated mainly in low- and middle-income countries, where structural barriers to timely diagnosis and treatment during prenatal care persist.^5^^,^^6^

Recent epidemiological analyses have also highlighted congenital syphilis as a re-emerging public health condition.^7^^,^^8^ Furthermore, evidence suggests that most adverse outcomes associated with congenital syphilis occur among women who were not adequately screened, diagnosed, or treated during prenatal care, reflecting preventable failures in maternal healthcare.^2^ Other factors associated with gestational and congenital syphilis include socioeconomic vulnerability, low maternal education, inadequate prenatal care, delayed diagnosis, failure to treat sexual partners, and maternal comorbidities such as HIV infection and diabetes mellitus.^4^^,^^7^, ^8^, ^9^ These determinants may increase the risk of vertical transmission and contribute to adverse neonatal outcomes, particularly in socially vulnerable settings.^6^, ^7^, ^8^

Population-based studies simultaneously evaluating factors associated with gestational syphilis and neonatal outcomes related to congenital syphilis remain limited, particularly in socially vulnerable contexts. Therefore, this study aimed to estimate the prevalence of gestational syphilis and the birth prevalence of congenital syphilis, identify factors associated with gestational syphilis, and evaluate the association between congenital syphilis and adverse neonatal outcomes in Guarujá, a harbor city in the São Paulo state, Brazil.

## Methods

### Study design, setting, and population

This cross-sectional study was nested within the population-based Guaru-yá Birth Cohort, entitled “Maternal and Child Health and Mortality in Guarujá”, which followed mother-infant pairs from birth to the first year of life. The study included all hospital births from mothers residing in the municipality of Guarujá occurring between April 15, 2024 and April 15, 2025.

Data collection was conducted in the two maternity hospitals located in the municipality, one public and one private. The public maternity hospital accounts for approximately 92% of births among women using the public healthcare system, indicating that most public-sector deliveries occur within the municipality. The private maternity hospital accounts for approximately 33% of births among women using private healthcare services, as a substantial proportion of private deliveries occur in neighboring municipalities. Therefore, the present study may be considered population-based for users of the public healthcare system in Guarujá.

### Data collection

Trained fieldworkers performed daily data collection in the two maternity hospitals of the municipality. Women were invited to participate within 48 h after delivery. After written informed consent was obtained, face-to-face interviews were conducted using a structured questionnaire, with an average duration of 20 min, collecting environmental, sociodemographic, lifestyle, obstetric, and health-related information. Data regarding delivery and neonatal outcomes were extracted from medical records, and photographs of prenatal care cards were obtained. All data were collected using forms developed in the Research Electronic Data Capture (REDCap) platform.^10^

### Variables

For the primary analysis, gestational syphilis was defined based on reactive treponemal and non-treponemal tests (VDRL) documented during pregnancy or at delivery admission, together with information on diagnosis and treatment during the current pregnancy. Women who had been adequately treated during pregnancy were not classified as cases of gestational syphilis.^1^^,^^11^ Exposure variables included maternal sociodemographic, lifestyle, and obstetric characteristics. Sociodemographic variables included maternal age (≤ 19, 20–34, and ≥ 35 years), maternal education (≤ 8, 9–11, and ≥ 12 years of schooling), skin color (white/non-white), cohabitation with a partner, paid employment, Bolsa Família benefit (a Brazilian government cash transfer program), household overcrowding (≤ 1 or > 1 resident per room), and wealth index terciles. Lifestyle variables included smoking, alcohol consumption, and illicit drug use during pregnancy. Obstetric variables included prenatal care attendance, prenatal care adequacy (adequate/inadequate), gravidity (primigravida/multigravida), and pregnancy planning.

Household overcrowding was defined according to the American Crowding Index as the ratio between household residents and rooms, with > 1 resident per room considered household overcrowding.^12^ Socioeconomic status was assessed using the wealth index proposed by Filmer and Pritchett,^13^ constructed through principal component analysis based on household assets and housing characteristics. Information on smoking, alcohol consumption, and illicit drug use during pregnancy was self-reported and verified using prenatal care cards. When discrepancies were identified, the behavior was considered present to minimize underreporting bias.

Prenatal care adequacy was operationally defined based on gestational age at the first prenatal visit and the number of visits completed according to gestational age at delivery, using the 2016 WHO antenatal care contact schedule as reference. Prenatal care was considered adequate when initiated by the 12^th^ gestational week and when the minimum number of visits expected for gestational age at delivery was reached: ≥ 1 visit for < 20 weeks, ≥ 2 for 20–< 26 weeks, ≥ 3 for 26–< 30 weeks, ≥ 4 for 30–< 34 weeks, ≥5 for 34–< 36 weeks, ≥ 6 for 36–< 38 weeks, ≥ 7 for 38–< 40 weeks, and ≥ 8 for ≥ 40 weeks. All other situations, including absence of prenatal care, were classified as inadequate.^14^

For the secondary analysis, neonatal outcomes included low birth weight (< 2500 g), prematurity (< 37 weeks), type of delivery (vaginal/cesarean), Apgar score at 1 min (< 7/≥ 7), neonatal care type (rooming-in, intensive care unit or intermediate care unit), fetal or neonatal death, and congenital malformations.

Congenital syphilis cases were defined as newborns from mothers diagnosed with syphilis during pregnancy or delivery who met at least one of the following criteria: absent or inadequate maternal treatment during pregnancy; neonatal non-treponemal test (VDRL) titer at least fourfold higher than the maternal titer at delivery; or clinical, laboratory, or radiological evidence compatible with congenital syphilis. Maternal treatment adequacy was defined according to national guidelines, considering completion of the stage-appropriate benzathine penicillin regimen, based on the documented number and timing of doses during pregnancy.^1^^,^^15^ All congenital syphilis cases were classified according to the official case definition and surveillance criteria established by the Brazilian Ministry of Health.^15^

### Statistical analysis

Descriptive analyses were performed using absolute and relative frequencies. Bivariate associations between independent variables and gestational syphilis, as well as between congenital syphilis and neonatal outcomes, were assessed using Pearson’s Chi-Square test.

Factors associated with gestational syphilis were analyzed using a hierarchical model based on the approach proposed by Victora et al.^16^ Distal variables included socioeconomic and demographic characteristics, intermediate variables included behavioral and reproductive characteristics during pregnancy, and proximal variables included prenatal care variables (Supplementary Fig. S1).

Associations were assessed using Poisson regression with robust variance, estimating Prevalence Ratios (PR) and 95% Confidence Intervals (95% CI). Variables were sequentially introduced according to the hierarchical model, with adjustment for variables from the same and preceding levels. Variables with p≤0.20 were retained at each stage, while variables included in the conceptual model remained in the final model regardless of statistical significance. Statistical significance was set at 5% in two-sided tests.

Multicollinearity was assessed using the Variance Inflation Factor (VIF), with no evidence of relevant collinearity. All analyses were performed using Stata version 13.0 (StataCorp LP, College Station, TX, USA).

## Results

Of the 2,294 eligible births during the study period, 2,217 were included in the analyses after exclusion of refusals (2.1%) and losses due to early hospital discharge (1.3%). The prevalence of gestational syphilis was 12.8% (95% CI: 11.4–14.2). Most participants were aged 20–34 years, non-white, had intermediate education, cohabited with a partner, had no paid employment, and did not receive Bolsa Família benefit.

Higher prevalence of gestational syphilis was observed among adolescents, women with low maternal education, non-white women, those not cohabiting with a partner, without paid employment, and among women receiving Bolsa Família benefit. Gestational syphilis was also more frequent among women living in household overcrowding conditions and those in the lowest wealth index tercile, indicating an important socioeconomic gradient in disease occurrence (Table 1).

**Table 1:** Socioeconomic and demographic characteristics by gestational syphilis status. Guaru-yá Birth Cohort, Guarujá, São Paulo, Brazil, 2024–2025.

Variable	Total (n = 2217)	No gestational syphilis (n = 1933)	Gestational syphilis (n = 284)	p-value
	n (%)	n (%)	n (%)	
Maternal age (years)				**0.005**
≤ 19	139 (6.3)	115 (82.7)	24 (17.3)	
20–34	1717 (77.4)	1486 (86.6)	231 (13.4)	
≥ 35	361 (16.3)	332 (92.0)	29 (8.0)	
Maternal education				**<0.001**
≤ 8 years	413 (18.7)	332 (80.4)	81 (19.6)	
9–11 years	1521 (68.6)	1335 (87.8)	186 (12.2)	
≥ 12 years	282 (12.7)	266 (94.3)	16 (5.7)	
Skin color				**0.008**
Non-white	1602 (72.3)	1378 (86.0)	224 (14.0)	
White	615 (27.7)	555 (90.2)	60 (9.8)	
Cohabitation with partner				**<0.001**
No	511 (23.0)	400 (78.3)	111 (21.7)	
Yes	1706 (77.0)	1533 (89.9)	173 (10.1)	
Paid employment				**<0.001**
No	1315 (59.6)	1122 (85.3)	193 (14.7)	
Yes	891 (40.4)	805 (90.4)	86 (9.6)	
Bolsa Família benefit				**0.004**
No	1561 (70.4)	1382 (88.5)	179 (11.5)	
Yes	656 (29.6)	551 (84.0)	105 (16.0)	
Household overcrowding				**0.039**
≤ 1 resident per room	1732 (79.7)	1528 (88.2)	204 (11.8)	
> 1 resident per room	441 (20.3)	373 (84.6)	68 (15.4)	
Wealth index terciles				**<0.001**
1st tercile (lowest)	739 (33.6)	611 (82.7)	128 (17.3)	
2nd tercile	787 (35.7)	694 (88.2)	93 (11.8)	
3rd tercile (highest)	677 (30.7)	621 (91.7)	56 (8.3)	

*Totals may differ due to missing data.

**Percentages represent gestational syphilis prevalence within each category of the independent variables.

Among the evaluated women, 18.4% reported smoking, 21.7% alcohol consumption, and 5.2% illicit drug use during pregnancy. Planned pregnancy was reported by 28.4% of participants. Nearly all women attended prenatal care, although nearly 30% had inadequate quantitative prenatal care (Table 2).

**Table 2:** Lifestyle habits during pregnancy and obstetric characteristics by gestational syphilis status. Guaru-yá Birth Cohort, Guarujá, São Paulo, Brazil, 2024–2025.

Variable	Total (n = 2217)	No gestational syphilis (n = 1933)	Gestational syphilis (n = 284)	p-value
	n (%)	n (%)	n (%)	
Smoking				**<0.001**
No	1810 (81.6)	1632 (90.2)	178 (9.8)	
Yes	407 (18.4)	301 (74.0)	106 (26.0)	
Alcohol consumption				**<0.001**
No	1736 (78.3)	1553 (89.5)	183 (10.5)	
Yes	480 (21.7)	379 (79.0)	101 (21.0)	
Illicit drug use				**<0.001**
No	2102 (94.8)	1861 (88.5)	241 (11.5)	
Yes	115 (5.2)	72 (62.6)	43 (37.4)	
Pregnancy planning				**<0.001**
No	1580 (71.6)	1352 (85.6)	228 (14.4)	
Yes	626 (28.4)	572 (91.4)	54 (8.6)	
Prenatal care				**<0.001**
No	31 (1.4)	19 (61.3)	12 (38.7)	
Yes	2186 (98.6)	1914 (87.6)	272 (12.4)	
Prenatal care adequacy				**<0.001**
Inadequate	688 (31.0)	568 (82.6)	120 (17.4)	
Adequate	1529 (69.0)	1365 (89.3)	164 (10.7)	
Gravidity				0.799
Primigravida	758 (34.2)	659 (86.9)	99 (13.1)	
Multigravida	1459 (65.8)	1274 (87.3)	185 (12.7)	

*Totals may differ due to missing data.

**Percentages represent gestational syphilis prevalence within each category of the independent variables.

Higher prevalence of gestational syphilis was observed among women who reported smoking, alcohol consumption, and illicit drug use during pregnancy. Gestational syphilis was also more frequent among women with unplanned pregnancy, those who did not attend prenatal care, and those with quantitatively inadequate prenatal care (Table 2).

In the adjusted hierarchical model (Table 3), low maternal education was associated with higher prevalence of gestational syphilis, with a prevalence 2.47 times that observed among women with ≤ 8 years of schooling compared with those with ≥ 12 years (95% CI: 1.43–4.29). Higher prevalence of gestational syphilis was also observed among women in the lowest wealth index tercile (PR = 1.46; 95% CI: 1.07–2.00) and those not cohabiting with a partner (PR = 1.83; 95% CI: 1.45–2.31). Conversely, women aged ≥ 35 years presented lower prevalence of gestational syphilis compared with those aged 20–34 years (PR = 0.67; 95% CI: 0.47–0.97).

**Table 3:** Crude and adjusted associations between socioeconomic, behavioral, and prenatal factors and gestational syphilis. Guaru-yá Birth Cohort, Guarujá, São Paulo, Brazil, 2024–2025.

Variable	Crude PR	95% CI	p-value	Adjusted PR	95% CI	p-value
**Distal determinants**						
Maternal age (years)						
≤ 19	1.28	0.86–1.88	0.202	0.90	0.60–1.34	0.596
20–34	Reference			Reference		
≥ 35	0.60	0.41–0.86	**0.006**	0.67	0.47–0.97	**0.033**
Maternal education						
≤ 8 years	3.46	2.07–5.78	**<0.001**	2.47	1.43–4.29	**0.001**
9–11 years	2.16	1.31–3.54	**0.002**	1.60	0.95–2.68	0.075
≥ 12 years	Reference			Reference		
Skin color						
White	Reference			Reference		
Non-white	1.43	1.10–1.88	**0.009**	1.22	0.94–1.61	0.138
Cohabitation with partner						
Yes	Reference			Reference		
No	2.14	1.72–2.66	**<0.001**	1.83	1.45–2.31	**<0.001**
Paid employment						
Yes	Reference			Reference		
No	1.52	1.20–1.93	**0.001**	1.21	0.95–1.55	0.122
Wealth index terciles						
1st tercile (lowest)	2.09	1.56–2.81	**<0.001**	1.46	1.07–2.00	**0.018**
2nd tercile	1.43	1.04–2.00	**0.027**	1.17	0.86–1.61	0.324
3rd tercile (highest)	Reference			Reference		
**Intermediate determinants**						
Pregnancy planning						
No	0.60	0.45–0.79	**<0.001**	0.81	0.61–1.09	0.166
Yes	Reference			Reference		
Smoking						
Yes	2.65	2.14–3.28	**<0.001**	1.75	1.35–2.27	**<0.001**
No	Reference			Reference		
Alcohol consumption						
Yes	2.00	1.60–2.49	**<0.001**	1.26	0.98–1.62	0.067
No	Reference			Reference		
Illicit drug use						
Yes	3.26	2.50–4.25	**<0.001**	1.70	1.26–2.28	<**0.001**
No	Reference			Reference		
**Proximal determinants**						
Prenatal care adequacy						
Adequate	Reference			Reference		
Inadequate	1.62	1.31–2.02	**<0.001**	1.18	0.94–1.47	0.147

Prevalence Ratios (PR) were estimated using Poisson regression with robust variance and respective 95% Confidence Intervals (95% CI). Crude models were obtained through bivariate analyses. Adjusted models were constructed using a hierarchical approach with sequential inclusion of distal, intermediate, and proximal determinants. “No response/unknown” categories were retained in the models exclusively for adjustment purposes and are therefore not presented.

At the intermediate level, smoking during pregnancy was associated with 75% higher prevalence of gestational syphilis (PR = 1.75; 95% CI: 1.35–2.27), while illicit drug use was associated with 70% higher prevalence of the outcome (PR = 1.70; 95% CI: 1.26–2.28). Although alcohol consumption, pregnancy planning, and prenatal care adequacy were associated with gestational syphilis in crude analyses, these associations lost statistical significance after adjustment (Table 3).

The birth prevalence of congenital syphilis was 5.8% (95% CI: 4.8–6.8). Among the evaluated newborns, 10.7% presented low birth weight, 12.6% were born preterm, and 2.0% resulted in fetal or neonatal death. Newborns with congenital syphilis presented higher frequency of low birth weight, prematurity, and fetal or neonatal death compared with those without congenital syphilis. No statistically significant associations were observed between congenital syphilis and the other evaluated neonatal outcomes (Table 4).

**Table 4:** Neonatal outcomes by congenital syphilis status. Guaru-yá Birth Cohort, Guarujá, São Paulo, Brazil, 2024–2025.

Variable	Total (n = 2217)	No congenital syphilis (n = 2088)	Congenital syphilis (n = 129)	p-value
	n (%)	n (%)	n (%)	
Low birth weight				**0.017**
No	1979 (89.3)	1872 (89.7)	107 (83.0)	
Yes	238 (10.7)	216 (10.3)	22 (17.0)	
Prematurity				**0.035**
No	1937 (87.3)	1832 (87.7)	105 (81.4)	
Yes	280 (12.6)	256 (12.3)	24 (18.6)	
Type of delivery				0.225
Vaginal	1157 (52.1)	1083 (51.9)	74 (57.4)	
Cesarean	1060 (47.8)	1005 (48.1)	55 (42.6)	
Apgar score at 1 min				0.275
≥ 7	2035 (92.7)	1925 (92.8)	110 (90.2)	
< 7	161 (7.3)	149 (7.2)	12 (9.8)	
Neonatal care type				0.327
Rooming-in	1974 (92.7)	1871 (92.8)	103 (90.4)	
Intensive or intermediate care unit	156 (7.3)	145 (7.2)	11 (9.7)	
Fetal or neonatal death				**<0.001**
No	2172 (98.0)	2053 (98.3)	119 (92.3)	
Yes	45 (2.0)	35 (1.7)	10 (7.8)	
Congenital malformations				0.186*
Absent	2147 (96.8)	2019 (96.7)	128 (99.2)	
Present	70 (3.2)	69 (3.3)	1 (0.8)	

⁎ Fisher’s exact test.

In the crude analyses, newborns with congenital syphilis presented higher prevalence of low birth weight (PR = 1.65; 95% CI: 1.10–2.46), prematurity (PR = 1.52; 95% CI: 1.04–2.16), and fetal or neonatal death (PR = 4.62; 95% CI: 2.34–9.13) compared with those without congenital syphilis. After adjustment, only the association with fetal or neonatal death remained statistically significant, with prevalence 3.25 times that observed among newborns without congenital syphilis (PR = 3.25; 95% CI: 1.74–6.07) (Table 5).

**Table 5:** Crude and adjusted associations between congenital syphilis and neonatal morbidity and mortality outcomes. Guaru-yá Birth Cohort, Guarujá, São Paulo, Brazil, 2024–2025.

Outcomes	Crude PR	95%CI	p-value	Adjusted PR	95%CI	p-value
Low birth weight						
Yes	1.65	1.10–2.46	**0.015**	1.33	0.88–2.01	0.184
No	Reference			Reference		
Prematurity						
Yes	1.52	1.04–2.16	**0.031**	1.28	0.86–1.90	0.225
No	Reference			Reference		
Apgar score at 1 min						
< 7	1.37	0.78–2.40	0.271	1.31	0.75–2.30	0.347
≥ 7	Reference			Reference		
Neonatal care type						
Intensive or intermediate care unit	1.34	0.75–2.53	0.240	1.19	0.64–2.21	0.582
Rooming-in	Reference			Reference		
Fetal or neonatal death						
Yes	4.62	2.34–9.13	**<0.001**	3.25	1.74–6.07	**<0.001**
No	Reference			Reference		

Prevalence Ratios (PR) were estimated using Poisson regression with robust variance and respective 95% Confidence Intervals (95% CI). Crude models were obtained through bivariate analyses. Adjusted models included maternal age, maternal education, cohabitation with a partner, prenatal care attendance, and presence of risk behaviors during pregnancy (tobacco, alcohol consumption, and/or illicit drug use).

## Discussion

The present study identified a high prevalence of gestational syphilis (12.8%) and a high birth prevalence of congenital syphilis (5.8%) in the municipality of Guarujá, showing a substantial burden of infection in an urban setting marked by social vulnerability. The findings demonstrated that social and behavioral determinants played a central role in disease occurrence, as evidenced by the adopted hierarchical model. In addition, congenital syphilis was associated with fetal or neonatal death, reinforcing the impact of vertical transmission on severe neonatal outcomes. Global evidence indicates that maternal syphilis remains responsible for hundreds of thousands of adverse outcomes annually, including stillbirth, prematurity, and preventable neonatal deaths, particularly in low- and middle-income countries.^2^^,^^17^

National data also indicate persistently high levels of gestational and congenital syphilis in Brazil. According to the Brazilian Ministry of Health, in 2023 the detection rate of syphilis in pregnant women was 34.0 cases per 1,000 live births (3.40%), while congenital syphilis incidence reached 9.9 cases per 1,000 live births (0.99%).^18^ Although these indicators derive from national epidemiological surveillance systems and are therefore not directly comparable to data obtained through systematic screening in population-based studies, the values identified in the Guaru-yá Birth Cohort were substantially higher. These differences should be interpreted with caution because the measures are based on different case definitions, denominators, and ascertainment methods.^18^

Findings from previous Brazilian studies also demonstrate lower prevalence than those observed in the present investigation. In the multicenter “Birth in Brazil” study, conducted with postpartum women hospitalized in different Brazilian regions in 2011‒2012, gestational syphilis prevalence was estimated at 1.02%.^19^ However, direct comparison should be interpreted with caution given the substantial time gap and changes in the epidemiology and case definitions of syphilis in Brazil. In settings marked by high social vulnerability, such as a study conducted in the Brazilian Amazon, gestational syphilis prevalence was 5.2%;^20^ comparability is also limited by differences in sampling strategy and the use of VDRL without confirmatory testing. Conversely, a recent investigation conducted in the state of Rio de Janeiro identified similar magnitudes, with gestational syphilis prevalence of 14.5% and congenital syphilis prevalence of 5.3%.^21^ Together, these findings suggest that some Brazilian urban settings may concentrate an extremely high burden of vertical syphilis transmission, particularly in territories marked by persistent social inequalities and limitations in prenatal care effectiveness.^21^

The high prevalence observed in the present study may also be partially explained by characteristics of the study setting and data collection strategy. Guarujá is a harbor city marked by important urban social vulnerability and persistent socioeconomic inequalities, factors previously associated with higher burden of gestational syphilis and vertical transmission.^6^, ^7^, ^8^, ^9^^,^^21^ Furthermore, the systematic daily data collection performed directly in maternity hospitals, combined with verification of prenatal records and laboratory tests, likely increased case detection and reduced underdiagnosis compared with studies based exclusively on routine surveillance data.^18^^,^^21^

Factors associated with gestational syphilis at the distal level reflected important dimensions of social vulnerability. The independent association between low maternal education and lower wealth index tercile suggests that socioeconomic inequalities play an important role in the persistence of infection, even after adjustment for behavioral and healthcare-related factors. Although previous Brazilian studies have already described an association between socioeconomic vulnerability and higher occurrence of gestational syphilis,^9^^,^^19^^,^^22^ the findings of the present investigation gain relevance due to the high prevalence observed in an urban setting marked by substantial social inequalities. International evidence also indicates that persistent structural barriers compromise effectiveness of strategies aimed at preventing vertical transmission, even in settings with expanded prenatal care coverage.^5^^,^^23^ In this context, gestational syphilis appears to reflect not only individual vulnerabilities, but also social inequalities that may compromise timely diagnosis, continuity of care, and effectiveness of vertical transmission prevention strategies. Although skin color was not independently associated with gestational syphilis after adjustment, its dichotomous classification as White or non-White may have obscured differences between specific skin-color groups. Previous national evidence has demonstrated substantial ethnoracial inequalities in maternal and congenital syphilis in Brazil.^9^

The lower prevalence of gestational syphilis among women aged ≥35 years observed in the present study may reflect greater social and reproductive stability, as well as stronger linkage to healthcare services. Previous evidence suggests that older women tend to present greater adherence to prenatal care and higher use of preventive services, whereas younger pregnant women frequently experience greater social vulnerability, higher exposure to risk behaviors, and increased likelihood of late diagnosis and inadequate treatment during pregnancy.^5^^,^^24^ In the Brazilian context, studies have also demonstrated greater prenatal screening coverage and follow-up among older women.^19^ These findings suggest that younger pregnant women may represent a priority group for early screening, prevention, and longitudinal prenatal follow-up strategies.

At the intermediate level, smoking and illicit drug use remained independently associated with gestational syphilis after hierarchical adjustment, suggesting that these behaviors may act as markers of greater social vulnerability and exposure to high-risk contexts. Although alcohol consumption was associated with gestational syphilis in crude analyses, this association lost statistical significance after adjustment, indicating possible influence of correlated socioeconomic and behavioral factors. Previous evidence demonstrates that substance use during pregnancy may be associated with lower continuity of prenatal care, greater exposure to vulnerable situations, and greater difficulty adhering to treatment, factors that may contribute to persistence of vertical syphilis transmission.^11^

The absence of an independent association between quantitative prenatal care adequacy and gestational syphilis after adjustment suggests that the number of consultations alone may not be sufficient to prevent occurrence of the infection. This finding indicates that aspects not directly evaluated in the present study, such as timely diagnosis, adequate clinical management, prompt maternal treatment, therapeutic management of sexual partners, and continuity of care throughout pregnancy, may play an important role in this context. Inadequate management of sexual partners remains an important challenge for interrupting the transmission chain and preventing maternal reinfection during pregnancy.^1^^,^^15^

In the present study, congenital syphilis remained independently associated with fetal or neonatal death after adjustment for potential confounding factors, highlighting the substantial impact of vertical transmission on severe neonatal outcomes. Although associations with low birth weight and prematurity were observed in crude analyses, these lost statistical significance after adjustment. Previous evidence demonstrates that treponemal infection during pregnancy is associated with severe adverse outcomes, including fetal and neonatal death, particularly in situations of late diagnosis or inadequate maternal treatment.^2^^,^^7^ The findings of the present study reinforce that many of these outcomes could be reduced through timely diagnosis and adequate management during prenatal care.

These findings should also be considered within ongoing efforts to eliminate mother-to-child transmission of syphilis. WHO global guidance, the Pan American Health Organization (PAHO) Elimination of Mother-to-Child Transmission Plus (EMTCT Plus) initiative, and Brazil’s national certification strategy emphasize antenatal testing and adequate treatment, surveillance, and quality maternal and child health services; in this context, identifying high-burden settings may help prioritize interventions and support progress toward elimination goals.^25^, ^26^, ^27^

Among the limitations of the present study, the cross-sectional design should be highlighted, as it does not allow definitive causal inference. However, most evaluated exposures, particularly socioeconomic and healthcare-related determinants, temporally preceded the outcome, supporting plausibility of the observed associations and reducing the likelihood of reverse causality. Moreover, information regarding maternal lifestyle habits, such as smoking, alcohol consumption, and illicit drug use during pregnancy, was obtained through self-report and is therefore subject to recall and social desirability bias. This potential bias was partially mitigated through cross-verification with prenatal care records, a strategy that may have contributed to greater data accuracy. In addition, information on maternal syphilis treatment was obtained from prenatal care cards and may have been affected by incomplete or inaccurate documentation, potentially leading to misclassification of treatment adequacy and gestational syphilis status. Detailed quantitative maternal VDRL titers were not consistently available across prenatal records, limiting analyses according to specific titer thresholds. Furthermore, the study did not evaluate information related to treatment and management of sexual partners, a factor recognized as highly relevant for preventing maternal reinfection and interrupting syphilis transmission during pregnancy.^1^^,^^15^ Although congenital syphilis remained independently associated with fetal or neonatal death, the relatively small number of deaths should be considered when interpreting the precision and magnitude of this estimate. Residual confounding by unmeasured maternal, behavioral, and healthcare-related factors cannot be excluded. Finally, the findings may have limited generalizability to populations with substantially different socioeconomic characteristics and healthcare access.

Among the strengths of the present study, its population-based design should be emphasized, with broad coverage of births occurring in the municipality and minimal sample losses, strengthening external validity and representativeness of the findings. In addition, systematic syphilis screening during all hospital admissions for delivery reduced the possibility of underdiagnosis and ensured greater accuracy in the identification of gestational and congenital syphilis cases. Finally, use of a hierarchical conceptual model allowed more consistent epidemiological interpretation of the associations by considering the causal organization of distal, intermediate, and proximal determinants, as recommended by Victora et al.^16^

The findings of the present study demonstrate high burden of gestational and congenital syphilis in an urban setting marked by social vulnerability, highlighting the central role of social and behavioral determinants in the persistence of the infection. The independent association between congenital syphilis and fetal or neonatal death reinforces the impact of vertical transmission on severe neonatal outcomes. Together, the findings suggest that strategies focused exclusively on expanding prenatal care coverage may be insufficient to address gestational and congenital syphilis. Strengthening early access to prenatal care, repeated syphilis screening, timely maternal treatment and management of sexual partners, professional training, and integration with epidemiological surveillance, combined with efforts to reduce social inequalities, may represent key strategies for reducing vertical transmission and neonatal morbidity and mortality in highly vulnerable settings.

## Ethics approval

The study was conducted in accordance with the Declaration of Helsinki and approved by the local Research Ethics Committee of Universidade do Oeste Paulista (CAAE: 77667124.0.0000.5515). Written informed consent was obtained from all participants prior to data collection.

## Declaration of generative AI and AI-assisted technologies in the manuscript preparation process

During the preparation of this work, the authors used ChatGPT (OpenAI) to support English language revision and improve textual clarity. The authors critically reviewed and edited all content generated by the tool and take full responsibility for the final version of the manuscript.

## Authors’ contributions

Conceptualization and study design: MRJA and MBM. Data collection: MRJA, DCS, MDORV, and RM. Methodology and data analysis: MRJA, LAAP, PN, DCS and MBM. Drafting of the manuscript: MRJA. Critical revision of the manuscript: LAAP, MDORV, RM, PN, DCS and MBM. Supervision and project administration: MBM.

## Funding

This research received no specific grant from funding agencies in the public, commercial, or not-for-profit sectors.

## Data availability statement

The datasets generated and analyzed during the current study are not publicly available due to ethical and privacy restrictions but are available from the corresponding author upon reasonable request.

## Conflicts of interest

The authors declare that they have no known competing financial interests or personal relationships that could have appeared to influence the work reported in this paper.
