# Supplementary material for: Gestational and congenital syphilis in a Brazilian harbor city: A population-based study of prevalence, determinants, and adverse neonatal outcomes
Source: Braz J Infect Dis. 2026 Sep 30;30(5):105969. doi: 10.1016/j.bjid.2026.105969 (PMC13653979; doi:10.1016/j.bjid.2026.105969)
Supplement: Supplementary file 1 [file mmc1.docx]

**BJID-D-26-00149_ Supplementary Material**

**Supplementary Figure S1** Hierarchical conceptual framework used for analysis of gestational syphilis determinants in the Guaru-yá Birth Cohort, Guarujá, São Paulo, Brazil, 2024–2025.
